# Supplementary material for: Cellular senescence drives age-dependent hepatic steatosis
Source: Nat Commun. 2017 Jun 13;8:15691. doi: 10.1038/ncomms15691 (PMC5474745; doi:10.1038/ncomms15691)
Supplement: Supplementary Information — Supplementary Figures. [file ncomms15691-s1.pdf]

a

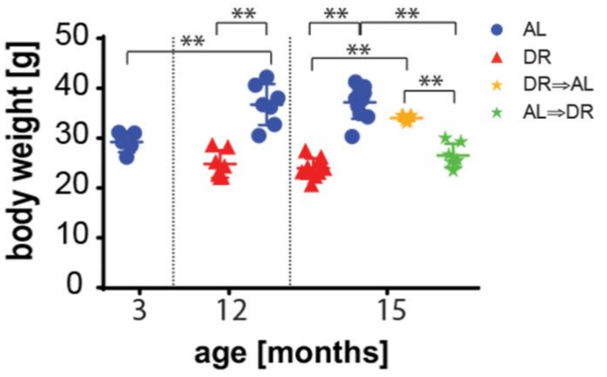

b

group	Case No	Grade of steatosis
15m AL	2746 NN	1
15m AL	2746 BN	1
15m AL	2638 2LN	3
15m AL	2638 2RN	3
12m AL	2640 RN	0
12m AL	2640 BN	1
12m AL	2640 LN	2
12m AL	2635 NN	2
12m DR	2629 RN	0
12m DR	2629 2RN	0
12m DR	2629 BN	0
12m DR	2629 LN	0
15m DR	2633 BN	0
15m DR	2633 2LN	1
15m DR	2633 LN	0
15m DR	2642 3W	0

Supplementary Figure 1:

a) Body weights of mice in all experimental groups**. b)** Steatosis scoring on H&E stained mouse liver sections at 12m and 15m of age (scores: 0 <5%, 1 = 5-33%, 2 = 34-66%, 3 = 67-100%).

Data per animal (dots) and means ± SD are shown. Mouse numbers for body weights are AL3m: n=5; AL/DR12m: n=7; AL/DR15m: n=10, crossover: n=7. Significant differences (ONE WAY ANOVA) are indicated with * P≤0.05 and ** P≤0.001.

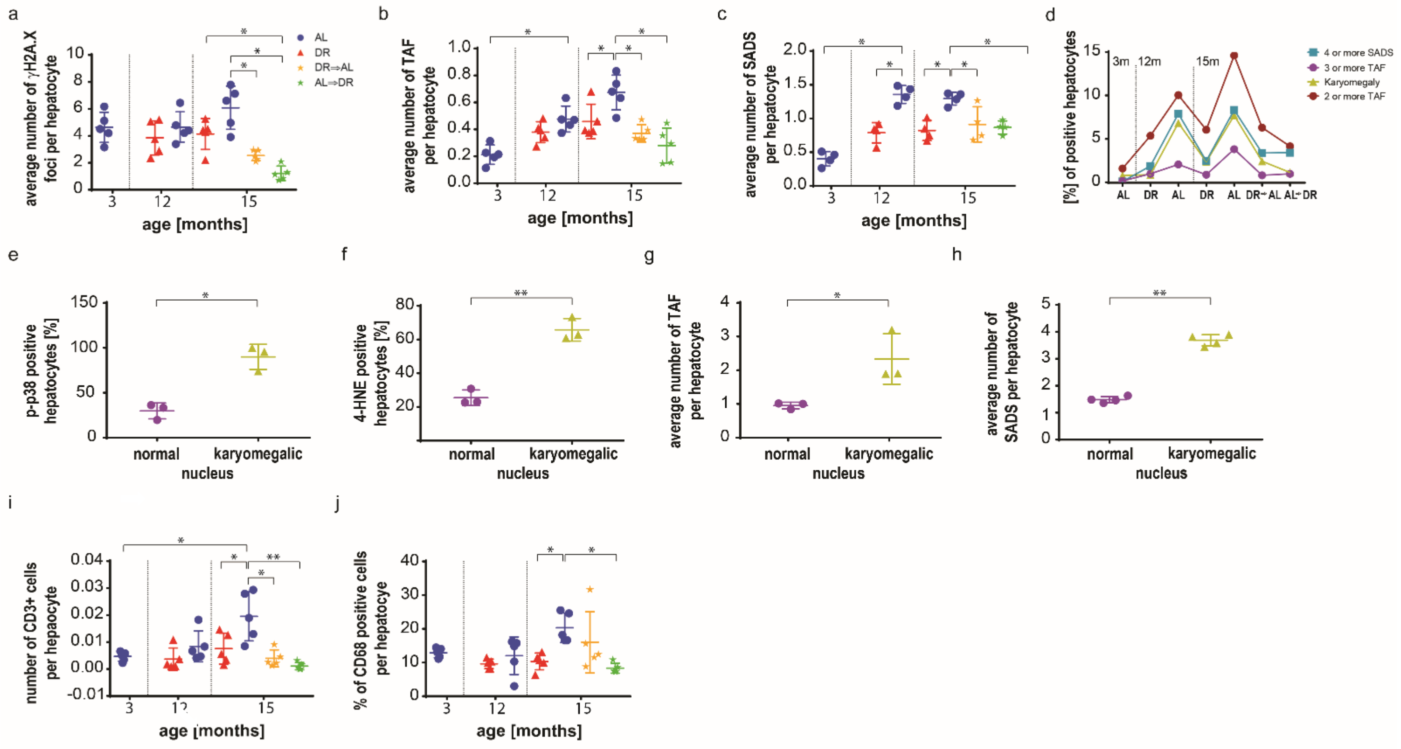

Supplementary Figure 2: Further markers of cell senescence in mouse livers

a) Average numbers of γ-H2A.X foci per hepatocyte (n=5). **b)** Average numbers of TAF per hepatocyte (n=5). **c)** Average numbers of SADS per hepatocyte (n=4). **d)** Plots of multiple senescence markers (percentage of karyomegalic hepatocytes, hepatocytes with ≥2 and ≥3 TAF or with ≥4 SADS) over all experimental groups show similar patterns. **e)** - **h)** Hepatocytes were separated into 'normal' (nuclear cross-sectional area <127 μm^2) and 'karyomegalic' (nuclear cross-sectional area ≥127 μm^2) **e)** frequencies of p38-positive cells in both groups (n=3), **f)** frequencies of 4-HNE positive hepatocytes (n=3), **g)** average numbers of TAF per hepatocyte nucleus (n=3), **h)** average numbers of SADS per hepatocyte nucleus (n=3), **i)** frequency of CD3+ cells per hepatocyte (n=5), **j)** frequency of CD68+ cells per hepatocyte (n=5).

Data per animal (dots) and means ± SD are shown. Significant differences (ONE WAY ANOVA, ttest) are indicated with * P≤0.05 and ** P≤0.001.

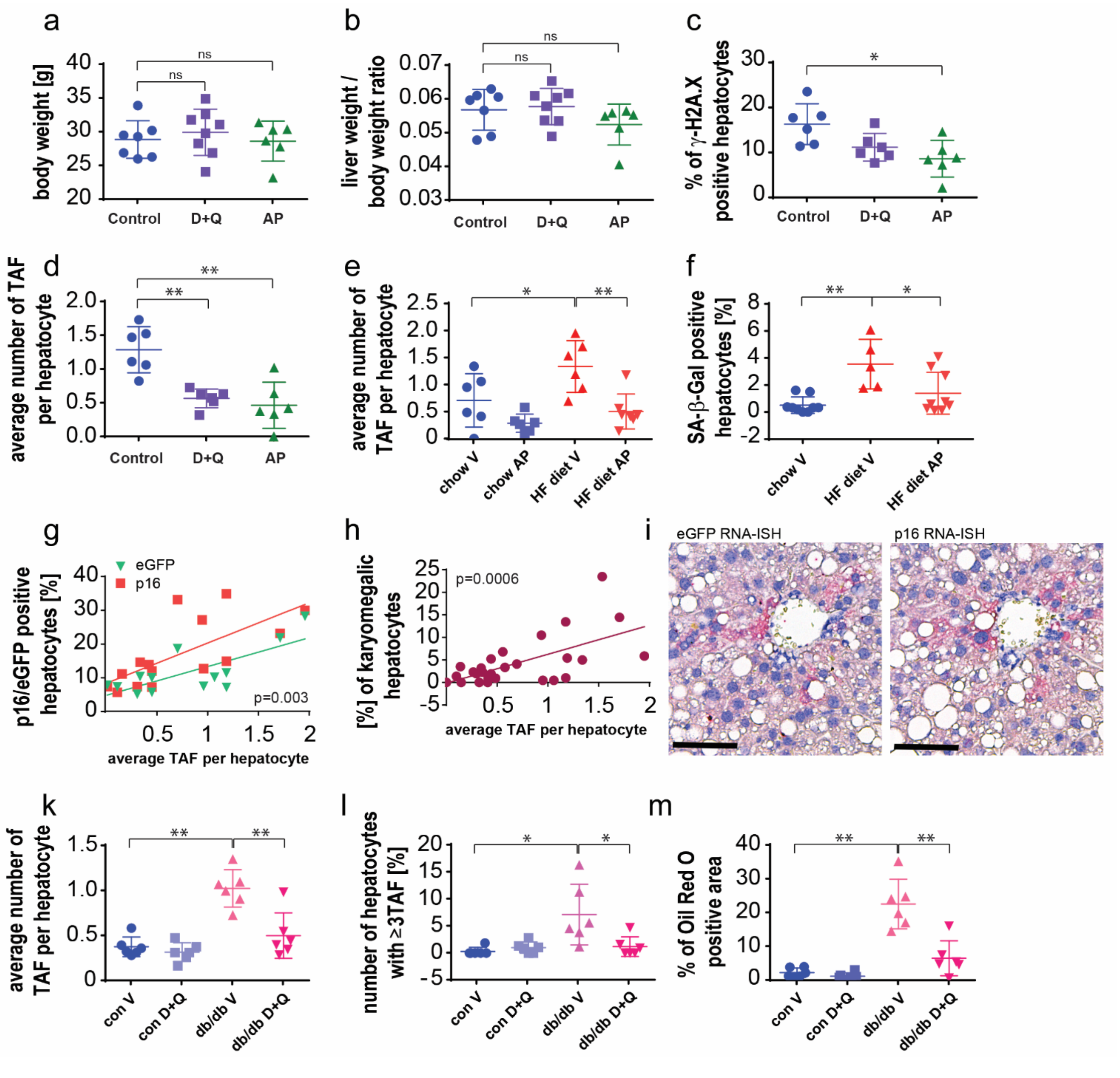

Supplementary Figure 3: Senescence marker after AP and senolytic treatment in old, high fat fed and db/db mice

a)-d) 27 months old INK-ATTAK mice show no differences in **a)** body weight (n=6-8) and **b)** liver/body weight ratio (n=6-8). **c)** Percentages of γ-H2A.X positive hepatocytes (n=6) and **d)** average numbers of TAF per hepatocyte decrease significantly after treatment with AP and senolytic drugs (n=6). **e)-i)** 15 months old INK-ATTAK mice under standard chow or high fat diet. **e)** Average number of TAF per hepatocyte (n=6) and **f)** number of SA-β-Gal positive hepatocytes increase in animals on high fat feeding and decrease significantly after treatment with AP (n=5-10). **g), h)** Showing correlations positive between the average number of TAF per hepatocyte with senescent markers p16/eGFP and karyomegaly. **i)** Representative images of RNA-ISH staining for p16 and eGFP on adjacent sections shows positive cells in the same area (red/pink = p16/eGFP, blue = haematoxylin, scale bar 50μm). **k)-m)** db/db mice **k)** Average number of TAF per hepatocyte (n=6), **l)** % of TAF^+ hepatocytes (n=6) and **m)** % of Oil Red O positive area are significantly increased in db/db animals and decreased significantly after treatment with senolytic drugs (n=6).

Data per animal (dots) and means ± SD are shown. Significant differences (ONE WAY ANOVA) are indicated with * P≤0.05 and ** P≤0.001.

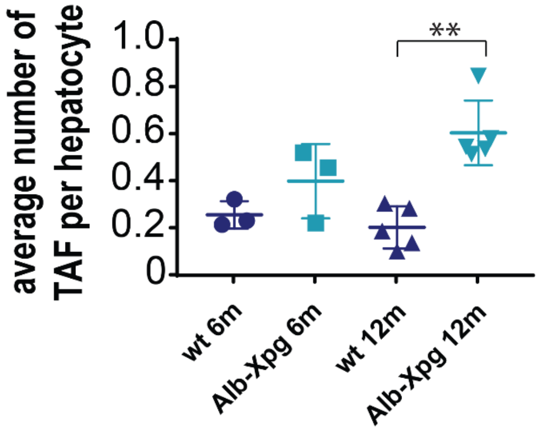

Supplementary Figure 4: Hepatocyte specific senescence leads to fat accumulation in liver

a) 6 (n=3) and 12 (n=5) months old *Alb-Xpg* mice show a significant increase in the average number of telomere associated foci (TAF) per hepatocyte.

All data are mean ± SD 3-5 animals per group. Significant differences (ONE WAY ANOVA) are indicated with * P≤0.05 and ** P≤0.001.

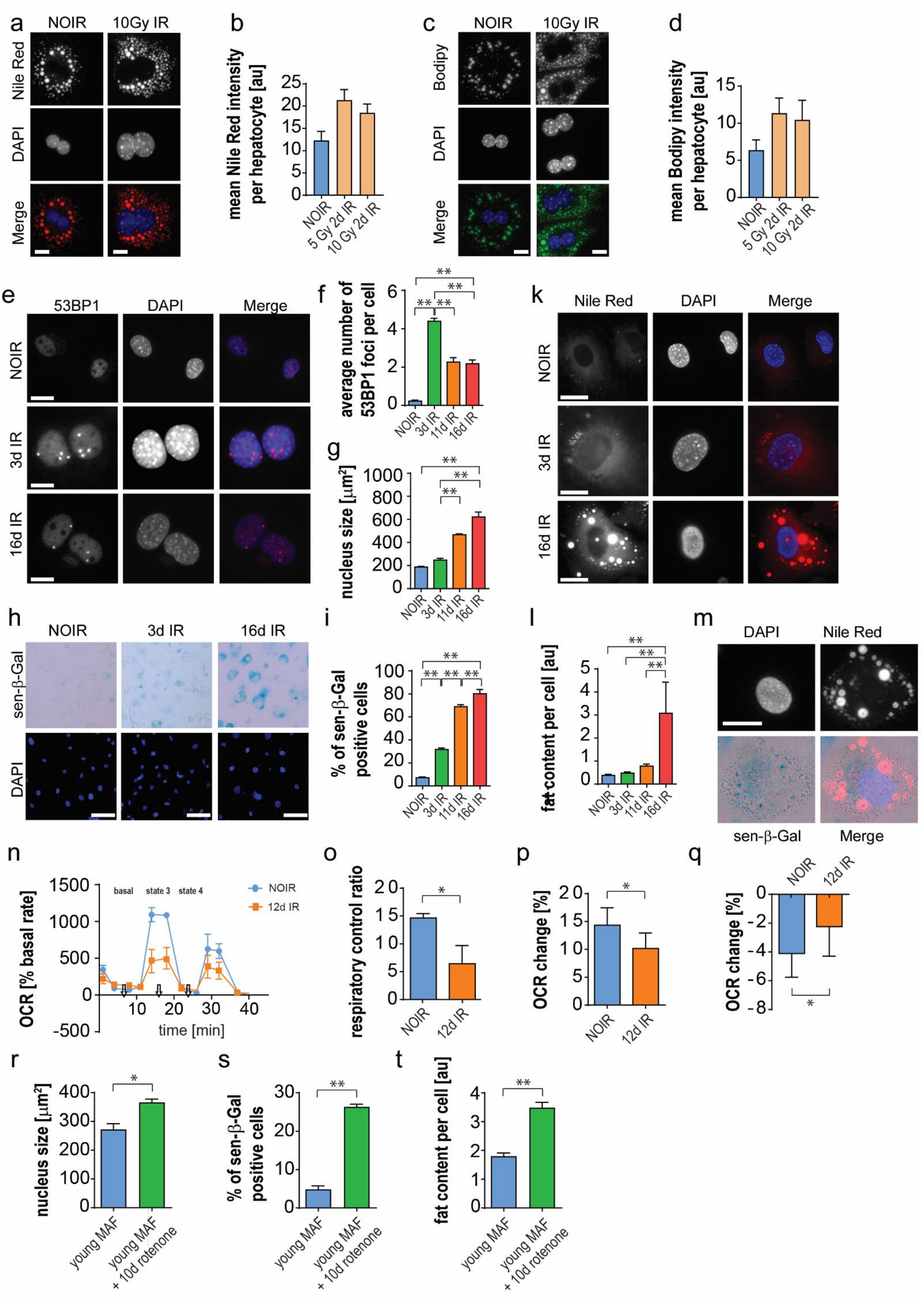
a
NOIR
10Gy IR
Nile Red
DAPI
Merge
b
mean Nile Red intensity per hepatocyte [au]
NOIR
5 Gy 2d IR
10 Gy 2d IR
c
Bodipy
d
mean Bodipy intensity per hepatocyte [au]
e
53BP1
3d IR
16d IR
f
average number of 53BP1 foci per cell
11d IR
g
nucleus size [µm²]
k
h
sen-β-Gal
i
% of sen-β-Gal positive cells
l
fat content per cell [au]
m
n
OCR [% basal rate]
basal
state 3
state 4
12d IR
time [min]
o
respiratory control ratio
p
OCR change [%]
q
r
young MAF
young MAF + 10d rotenone
s
t

Supplementary Figure 5: Induction of senescence causes mitochondrial dysfunction and reduces fatty acid oxidation capacity, resulting in fat accumulation in senescent cells

Fluorescent staining for Nile Red and BODIPY® 493/503 show similar staining patterns as well as a similar increase in staining after IR. **a, c)** Representative images showing Nile Red and BODIPY® 493/503 staining in hepatocytes after NOIR or 2 days after 5/10 Gy IR. (Merge: blue=DAPI, red=Nile Red (scale bar 20μm), green= BODIPY® 493/503 (scale bar 10μm), **b, d)** mean Nile red and BODIPY® 493/503 intensity show a similar increase in fat content per hepatocyte after IR. Mouse adult ear fibroblasts (MAF) acquire a senescent phenotype over one to two weeks after 10Gy IR: **e)** representative images showing 53BP1 DNA damage foci in non-irradiated MAFs (NOIR) and at 3 and 16 days after IR, Merge: blue=DAPI, red =53BP1 (scale bar=10μm)**, f)** frequencies of 53BP1 foci, **g)** nuclear size, **h)** representative images showing Sen-β-Gal staining in NOIR, 3 and 16 days after IR (scale bar=50μm), **i)** frequencies of Sen-β-Gal positive cells, **k)** Representative images showing Nile Red staining in NOIR, 3 and 16 days after IR. Merge: blue=DAPI, red =Nile Red, scale bar=20μm**, l)** Average fluorescent intensity of Nile Red staining in MAFs before and after irradiation. **m)** Sen-β-Gal positive MAF show accumulation of lipids (Merge: blue=DAPI, red=Nile Red, light blue=sen-β-Gal, scale bar=10μm)**, n)** A representative experiment showing oxygen consumption rates (OCR) from permeabilised MAFs without irradiation or at 12 days after IR, **o)** Respiratory control ratios were calculated as OCR at state 3 (in the presence of ADP) divided by state 4 (in the presence of oligomycin). **p)** Change in OCR after addition of the fatty acid palmitate as substrate, **q)** Change in OCR after inhibition of fatty acid oxidation by etomoxir. MAF were treated with rotenone (100nM) for 10days and showed an increase **r)** in nucleus size, **s)** the percentage of Sen-β-Gal positive cells and **t)** in the average fluorescence intensity of Nile Red staining.

Data b, d, i, r-t are mean ± SEM and f-l, n-q are SD with 3-4 animals per group. Significant differences (ONE WAY ANOVA) are indicated with * P≤0.05 and ** P≤0.001.

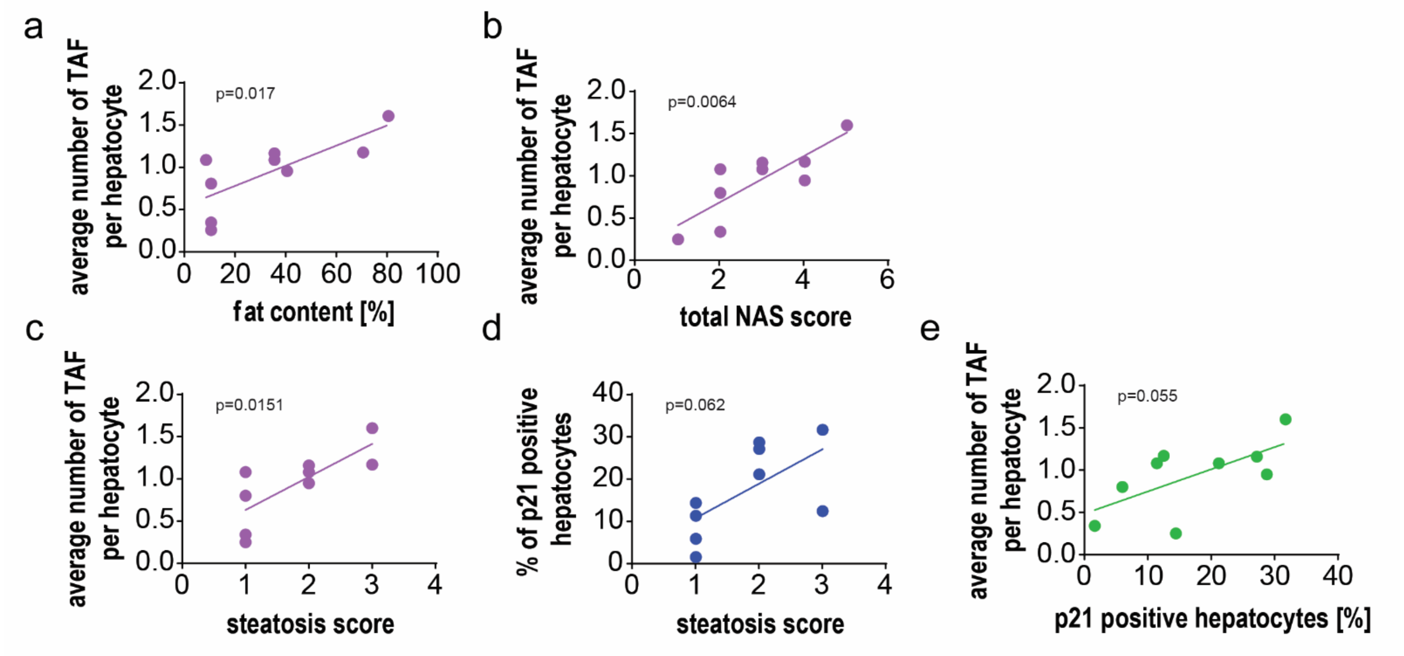

Supplementary Figure 6: Correlations between additional senescence markers and NAFLD scores in patients.

Markers of senescence increase with NAFLD score. **a)** Correlation between the average number of TAF per hepatocyte and fat content (R^2=0.5805) **b)** Correlation between the average number of TAF per hepatocyte and NAS score (R^2=0.6778), **c)** Correlation between the average number of TAF per hepatocyte and steatosis score of NAFLD patients (R^2=0.5938), **d)** weak correlation between frequency of p21 positive hepatocytes and steatosis score of NAFLD patients (R^2=0.4218). **e)** Weak correlation between the average number of TAF per hepatocyte and frequency of p21 positive hepatocytes per patient (R^2=0.4287).

All data are means (frequencies of TAF per hepatocyte, p21+ cells) or scores per patient, n=9.
